# Structural Basis of the 9-Fold Symmetry of Centrioles

**DOI:** 10.1016/j.cell.2011.01.008

**Published:** 2011-02-04

**Authors:** Daiju Kitagawa, Ioannis Vakonakis, Natacha Olieric, Manuel Hilbert, Debora Keller, Vincent Olieric, Miriam Bortfeld, Michèle C. Erat, Isabelle Flückiger, Pierre Gönczy, Michel O. Steinmetz

**Affiliations:** 1 Swiss Institute for Experimental Cancer Research (ISREC), School of Life Sciences, Swiss Federal Institute of Technology (EPFL), CH-1015 Lausanne, Switzerland; 2 Swiss Light Source, Paul Scherrer Institut, 5232 Villigen PSI, Switzerland; 3 Biomolecular Research, Paul Scherrer Institut, 5232 Villigen PSI, Switzerland; 4 Department of Biochemistry, University of Oxford, Oxford OX1 3QU, UK

## Abstract

The centriole, and the related basal body, is an ancient organelle characterized by a universal 9-fold radial symmetry and is critical for generating cilia, flagella, and centrosomes. The mechanisms directing centriole formation are incompletely understood and represent a fundamental open question in biology. Here, we demonstrate that the centriolar protein SAS-6 forms rod-shaped homodimers that interact through their N-terminal domains to form oligomers. We establish that such oligomerization is essential for centriole formation in *C. elegans* and human cells. We further generate a structural model of the related protein Bld12p from *C. reinhardtii*, in which nine homodimers assemble into a ring from which nine coiled-coil rods radiate outward. Moreover, we demonstrate that recombinant Bld12p self-assembles into structures akin to the central hub of the cartwheel, which serves as a scaffold for centriole formation. Overall, our findings establish a structural basis for the universal 9-fold symmetry of centrioles.

## Graphical abstract

**Graphical Abstract:**

**Figure:**
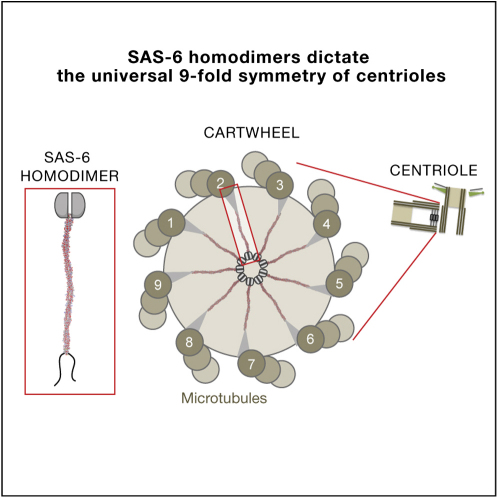

**Highlights:**

► Structural analysis of *C. elegans* centriolar protein SAS-6 reveals rod-shaped homodimers ► Higher-order oligomerization of SAS-6 homodimers is essential for centriole formation ► Nine homodimers of C. *reinhardtii* SAS-6 homolog Bld12p self-assemble into a ring ► Recombinant Bld12p forms structures akin to the hub and spokes of centriolar cartwheel

## Introduction

Centrioles are fundamental for the assembly of cilia and flagella across eukaryotic evolution (reviewed in Azimzadeh and Marshall, 2010). In addition, centrioles are important for assembling the centrosome, the major microtubule organizing center (MTOC) of animal cells, and as such, they are critical for genome stability. As anticipated from these important roles, aberrations in centriole structure or function are implicated in a number of disease conditions, including ciliopathies, male sterility, primary microcephaly, and cancer (reviewed in Nigg and Raff, 2009). Therefore, increased understanding of centriole biology is expected to also result in important clinical implications.

Centrioles, and the related basal bodies, are barrel-shaped microtubule-containing structures characterized by a universal 9-fold radial symmetry that they also impart to cilia and flagella (reviewed in Azimzadeh and Marshall, 2010). In most species, the centriole is organized around a cartwheel that comprises a central hub ∼25 nm in diameter from which nine spokes radiate outward and connect to nine microtubule blades (reviewed in Strnad and Gönczy, 2008). The molecular and structural principles directing the universal 9-fold symmetry of the cartwheel and the centriole remain to be discovered.

The genetic material duplicates once and only once per cell cycle, and so do centrioles. In contrast to the mechanisms governing DNA replication, however, those at the root of centriole formation are poorly understood. This is despite the fact that five proteins that are essential for centriole formation have been identified initially in *Caenorhabditis elegans* (Dammermann et al., 2004; Delattre et al., 2004; Kemp et al., 2004; Kirkham et al., 2003; Leidel et al., 2005; Leidel and Gönczy, 2003; O'Connell et al., 2001; Pelletier et al., 2004). Relatives of these components are present and similarly required for centriole formation across eukaryotic evolution, indicating that they constitute an ancient core module that is essential for centriole formation (reviewed in Nigg and Raff, 2009; Strnad and Gönczy, 2008).

Among these five components, SAS-6 is of particular interest to consider for investigating the mechanisms governing centriole formation for a number of reasons. First, proteins of the SAS-6 family are required for the earliest steps of centriole formation from *Chlamydomonas reinhardtii* to *Homo sapiens* (Culver et al., 2009; Leidel et al., 2005; Nakazawa et al., 2007; Rodrigues-Martins et al., 2007; Strnad et al., 2007; Yabe et al., 2007). Second, overexpression of SAS-6 proteins induces the formation of multiple new centrioles adjacent to the existing one in human cells (Strnad et al., 2007), as well as centriole amplification and de novo formation in *Drosophila melanogaster* (Rodrigues-Martins et al., 2007). Furthermore, combined overexpression in *Drosophila* spermatocytes of DSas-6 and the interacting protein Ana2 results in the formation of structures that resemble the cartwheel (Stevens et al., 2010). Third, SAS-6 proteins localize to the cartwheel in *C. reinhardtii* and *Tetrahymena thermophila* (Kilburn et al., 2007; Nakazawa et al., 2007), to the proximal part of the new centriole in *H. sapiens* (Kleylein-Sohn et al., 2007; Strnad et al., 2007), and to the functionally related central tube in *C. elegans* (Dammermann et al., 2008; Pelletier et al., 2006). Together, these observations suggest that proteins of the SAS-6 family are somehow important for the onset of centriole formation, although whether they can initiate this process on their own or must rely on additional factors to do so is not known. Overall, although it has been hypothesized that SAS-6 proteins may be critical for forming the central hub of the cartwheel (Strnad and Gönczy, 2008), the actual mechanisms by which they ensure cartwheel assembly and thus centriole formation have remained elusive.

In this study, using a combination of biophysical, biochemical, structural, and cell biological approaches, we establish that self-assembly of SAS-6 homodimers is at the root of the universal 9-fold symmetry of the cartwheel and thus of centrioles.

## Results

### Structural and Biophysical Characterization of *C. elegans* SAS-6

**Figure 1:**
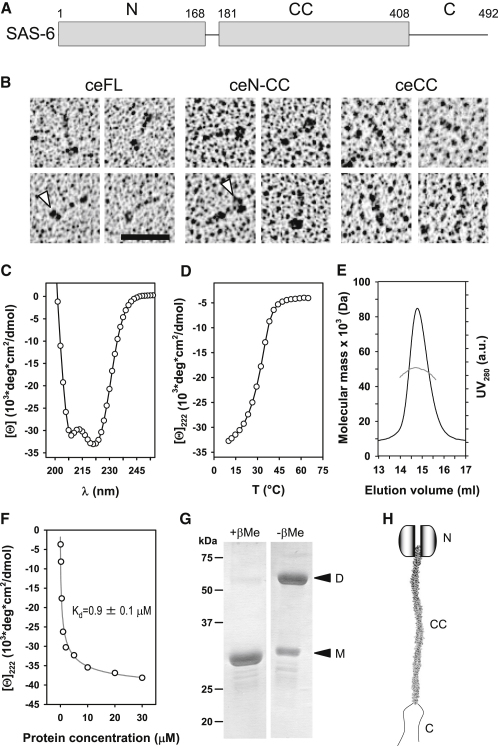
Domain Organization and Molecular Model of *C. elegans* SAS-6 (A) Schematic representation of *C. elegans* SAS-6. N, N-terminal domain; CC, coiled coil; C, C terminus. Numbers above the schematic correspond to amino acids. (B) Rotary metal shadowing electron micrographs of ceFL, ceN-CC, and ceCC specimens. Arrowheads indicate globular domains. Scale bar, 50 nm. (C and D) CD spectrum (C) and thermal unfolding profile recorded by CD at 222 nm (D) of the ceCC fragment. The data support the formation of a highly helical structure with moderate thermal stability. (E) MALS analysis of the ceCC fragment. The UV absorbance profile of size exclusion chromatography (black line) is overlaid with the molecular weight (50 kDa) estimation by MALS (gray line). (F) ceCC dilution series monitored by CD at 222 nm. The gray solid line represents the fit to the data (open circles) using a monomer-dimer model. (G) SDS-PAGE of the ceCC fragment run under reduced (+βMe) and nonreducing (−βMe) conditions. Arrowheads point to protein bands corresponding to monomeric (M) and disulfide-linked dimeric (D) forms of ceCC. (H) Molecular model of SAS-6 homodimer. Each monomeric subunit is composed of a globular N-terminal domain, a coiled-coil domain that forms a parallel dimer, and a poorly structured C-terminal part. See also Figure S1.

**Figure S1:**
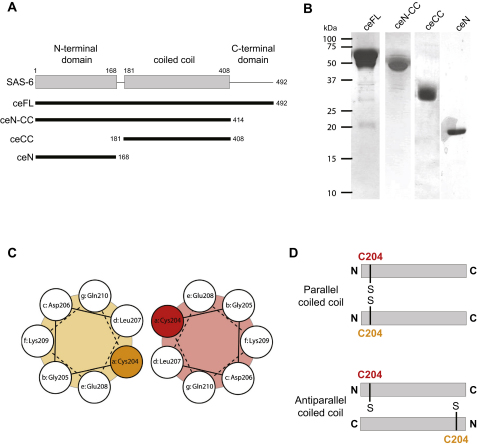
*C. elegans* SAS-6 Characterization, Related to Figure 1 (A) Schematic representation of *C. elegans* SAS-6 and fragments used in this study. ceFL: full-length (amino acids 1–492), ceN-CC: N-terminus plus coiled coil (amino acids 1–414), ceCC: coiled coil (amino acids 181–408); ceN: N-terminus (amino acids 1–168). (B) Sections of reducing and Coomassie-stained SDS-PAGE showing final purification products for the indicated recombinant proteins. From left to right: SAS-6 full-length (ceFL), ceN-CC (residues 1–414), ceCC (residues 181–408) and ceN (residues 1–168). Approximate molecular weights from in-gel markers are shown. (C) Helical wheel representation of the SAS-6 coiled-coil domain in the vicinity of Cys204 in a two-stranded parallel configuration. The predicted heptad repeat (denoted a to g) and the residues occupying its position are indicated. (D) Relative location of the Cys204 sulfur group on ceCC for a parallel or antiparallel coiled coil configuration. Efficient disulphide bridge formation is possible only in the parallel in-register coiled coil configuration.

We first set out to characterize the structure of *C. elegans* SAS-6 to uncover the mechanisms by which it contributes to centriole formation. Proteins of the SAS-6 family comprise an N-terminal domain with the evolutionarily conserved PISA motif, followed by a segment with a predicted coiled coil and a less-conserved C-terminal region predicted to be disordered (Figure 1A). We expressed and purified soluble SAS-6 full-length (ceFL), the N terminus plus the coiled coil (ceN-CC), or the coiled-coil domain alone (ceCC) (Figure 1A and Figures S1A and S1B available online) and analyzed them by biophysical and structural methods. Inspection by electron microscopy revealed an ∼35 nm elongated rod in all three constructs, which fits the predicted length of the SAS-6 coiled coil (∼220 residues × 0.1485 nm [axial raise per residue] = ∼32.7 nm) (Figure 1B). Full-length SAS-6 and ceN-CC were decorated with a globular head-like moiety at one end (Figure 1B, arrowheads), which is absent in ceCC, indicating that it corresponded to the N-terminal domain of SAS-6. No significant difference could be observed between ceFL and ceN-CC (Figure 1B), supporting the prediction that the C terminus does not adopt a globular structure.

We analyzed the ceCC fragment further to uncover its stability and molecular architecture. Circular dichroism (CD) spectroscopy revealed a far-ultraviolet spectrum and a cooperative thermal unfolding profile that is characteristic of moderately stable α-helical coiled-coil structures (Figures 1C and 1D) (Steinmetz et al., 1998). To assess the oligomerization state of the coiled-coil domain, we conducted multiangle light scattering (MALS) experiments, which yielded a molecular mass that is consistent with a dimer (50 kDa versus a ceCC monomeric mass of 27.4 kDa; Figure 1E). The stability of the ceCC coiled-coil dimer was estimated by measuring the change in CD signal at 222 nm upon dilution. Fitting of the data revealed a dissociation constant, K_d_, of 0.9 ± 0.1 μM (Figure 1F). To determine the relative orientation of the two ceCC monomers within the dimer, we performed SDS-PAGE analysis under nonreducing conditions. Cys204 is the only cysteine residue in the coiled coil and is predicted to occupy a heptad a core position, such that the ceCC fragment should form a disulphide bond only if the two fragments are in a parallel and in-register configuration (Figures S1C and S1D). As shown in Figure 1G, ceCC was indeed crosslinked under nonreducing conditions, indicating a parallel arrangement of monomers in SAS-6 homodimers (Figure 1H).

**Figure 2:**
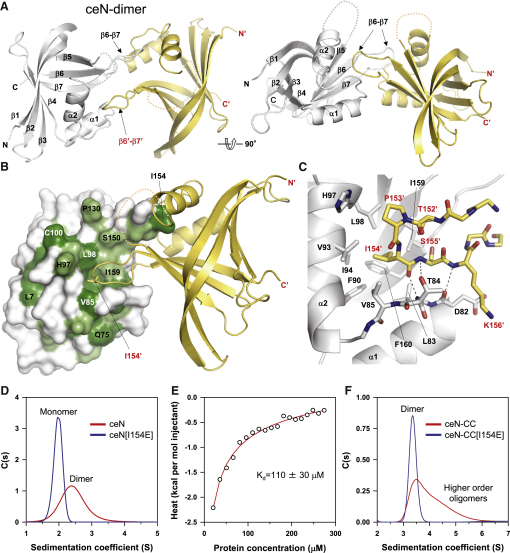
Structural Analysis of *C. elegans* SAS-6 N-Terminal Domain (A) Two overall views of the ceN-dimer structure seen in the asymmetric unit of the crystal 90° apart. Monomers A and B (in cartoon representation) are colored in light gray and yellow, respectively. Secondary structure elements and the N and C termini are assigned. Loop α2-β5, which is unique to *C. elegans*, is not seen in the electron density presumably due to disorder and is indicated by a dashed line. Each monomer displays two α helices that cap the end of a two-stranded β sheet sandwich. The PISA motif spans region β3 to α2, with evolutionarily conserved residues in this region contributing to the protein core as well as to a predominantly hydrophobic cavity between α1 and α2 (see also Figure S2). The locations of loops β6-β7 are indicated by arrows. (B) Structure of the ceN-dimer, with monomer A shown as surface representation. Highly conserved residues are colored dark green, and mostly conserved residues are colored bright green. I154 of monomer B is depicted as stick representation. (C) Close-up views of the interaction network observed at the dimer interface in cartoon (main chains) and stick (contacting residues) representations. Oxygen and nitrogen atoms are colored in red and blue, respectively, and carbon atoms are colored in light gray (monomer A) or yellow (monomer B). (D) Sedimentation velocity AUC analysis of ceN (red) and ceN[I154E] (blue) fragments. The peak labeled “Monomer” corresponds to a molecular weight of ∼20 kDa, which is consistent with the molecular weight of the ceN[I154E] monomer. The peak labeled “Dimer” corresponds to a molecular weight of ∼40 kDa. Protein concentration was 300 μM. (E) Dissociation isotherm obtained by ITC for ceN. A 1.6 mM ceN solution was injected stepwise into buffer. Shown are the integrated heat changes upon dilution. The solid red line represents the fit to the data (open circles) assuming dissociation of ceN dimers into monomers. (F) Sedimentation velocity AUC analysis of ceN-CC (red) and ceN-CC[I154E] (blue) fragments. The peak labeled “Dimer” corresponds to a molecular weight of ∼90 kDa, which is consistent with the formation of ceN-CC[I154E] dimers. The broad profile observed for ceN-CC (labeled “Higher-order oligomers”) suggests formation of higher-order oligomers beyond dimers. Protein concentration was 200 μM. See also Figure S2 and Figure S3.

**Figure S2:**
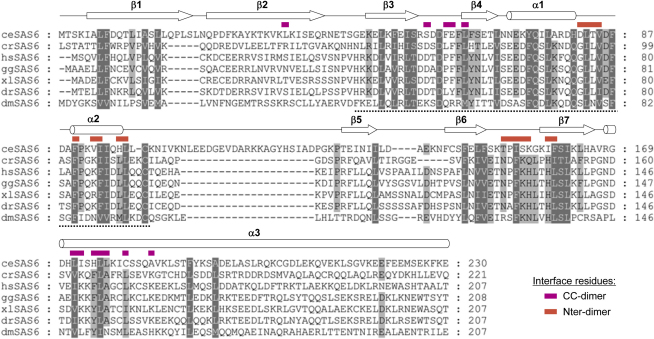
Structure-Based Sequence Alignment of SAS-6 Orthologs, Related to Figure 2 and Figure 4 Highly conserved and conserved residues are highlighted in dark and light gray, respectively. Secondary structure assignments based on the crystal structures of *C. elegans* SAS-6 and Bld12p (Figures 2 and 4) are shown on top of the alignment. Interacting residues seen in the CC-dimer are indicated in magenta; the ones seen in the N-dimer are indicated in red. The PISA domain characteristic of SAS-6 proteins is indicated by a dashed black line at the bottom of the alignment. Species identifiers are: ce, *Caenorhabditis elegans*; hs, *Homo sapiens*; gg, *Gallus gallus*; xl, *Xenopus laevis*; dr, *Dario rerio*; dm, *Drosophila melanogaster*; cr, *Chlamydomonas reinhardtii.* UniProtKB/Swiss-Prot sequence accession identifiers are as follows: ceSAS-6, SAS6_CAEEL; Bld12p (crSAS-6), A9CQL4_CHLRE; hsSAS-6, SAS6_HUMAN; ggSAS-6, SAS6_CHICK; xlSAS-6, SAS6_XENLA; drSAS-6, SAS6_DANRE; dmSAS-6, SAS6_DROME.

**Figure S3:**
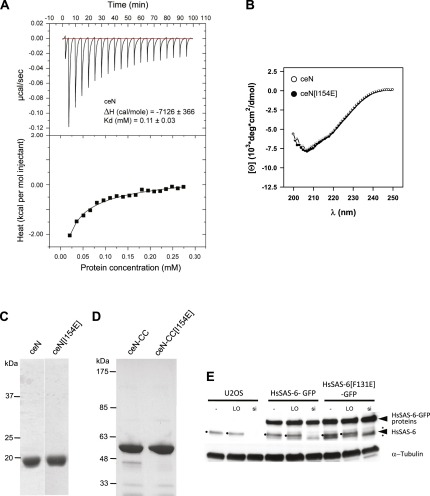
Characterization of *C. elegans* SAS-6 Protein Fragments and Depletion of Endogenous HsSAS-6 Using siHsSAS-6-3′UTR, Related to Figure 2 and Figure 3 (A) ITC of the *C. elegans* N-N interaction. Top panel: raw data representing the response to injections of ceN at high concentration into sample buffer. Bottom panel: integrated heat change (closed squares) and associated curve fit (black solid line). (B) CD spectrum of ceN (open circles) or ceN[I154E] (closed circles) fragments. (C) SDS-PAGE showing final purification products for AUC of ceN and ceN[I154E] recombinant proteins. (D) SDS-PAGE showing final purification products for AUC of ceN-CC and ceN-CC[I154E] recombinant proteins. (E) Cells left untreated (-), transfected with LO negative control siRNA (LO) or siHsSAS-6-3′UTR (si) for 48h before Western blot analysis with HsSAS-6 antibody; tubulin served as loading control. Arrows point to endogenous HsSAS-6 or HsSAS-6-GFP proteins. Dots indicate endogenous HsSAS-6 bands, stars unspecific bands.

Next, we determined the structure of the N-terminal globular domain of *C. elegans* SAS-6 (ceN; Figures S1A and S1B) by X-ray crystallography. We obtained crystals of a ceN variant and solved its structure to 2.1 Å resolution (Table S1). The asymmetric unit of the crystal contained a dimer of ceN monomers with local 2-fold symmetry (ceN-dimer) (Figure 2A). The fold of ceN is reminiscent of that of the XRCC4 family of DNA repair proteins (Junop et al., 2000). We noted that a striking interaction interface in the ceN-dimer was mediated by I154 at the tip of the β6-β7 loop of one monomer, which was inserted deeply into a hydrophobic cavity of the second monomer (Figures 2B and 2C). Both I154 and the residues shaping the hydrophobic cavity are well conserved among SAS-6 orthologs (Figure 2B and Figure S2), suggesting functional relevance. Analytical ultracentrifugation (AUC) experiments conducted at 300 μM protein concentration demonstrated that the ceN fragment could also form a dimer in solution (Figure 2D). Isothermal titration calorimetry (ITC) experiments yielded a K_d_ for the N-N interaction of ∼110 ± 30 μM (Figure 2E and Figure S3A), two orders of magnitude higher than that of the ceCC coiled coil. To address whether I154 mediates ceN-dimer formation, we substituted this residue for the charged residue glutamate (ceN[I154E]). Although the conformation of the domain was not altered by this mutation (Figures S3B and S3C), AUC experiments revealed that this change abrogated dimer formation (Figure 2D). We conclude that I154 is critical for mediating the N-N interaction.

Interestingly, inspection of the ceN-dimer structure suggested that the β6-β7 loop encompassing I154 might promote an interaction between SAS-6 homodimers, as this residue is located diametrically across the ceN domain's C terminus, which proceeds into the coiled coil (Figure 2A). To test this hypothesis, we conducted AUC experiments with the ceN-CC fragment, which could be more readily expressed and purified in an intact form than ceFL (Figure S3D). AUC of ceN-CC conducted at 200 μM protein concentration revealed the presence of higher-order oligomers besides dimers (Figure 2F). In contrast, a mutant in which I154 had been exchanged by glutamate (ceN-CC[I154E]) only formed dimers (Figure 2F).

Together, our structural and biophysical data establish that assembly of higher-order SAS-6 oligomeric structures occurs in two steps. First, elongated SAS-6 homodimers assemble, driven by the strong interaction between the helices of the two-stranded parallel coiled coil. Second, oligomers of SAS-6 homodimers assemble, a step that is mediated by the weaker interaction between pairs of N-terminal globular domains located in adjacent homodimers.

### Biological Significance of SAS-6 Oligomerization

**Figure 3:**
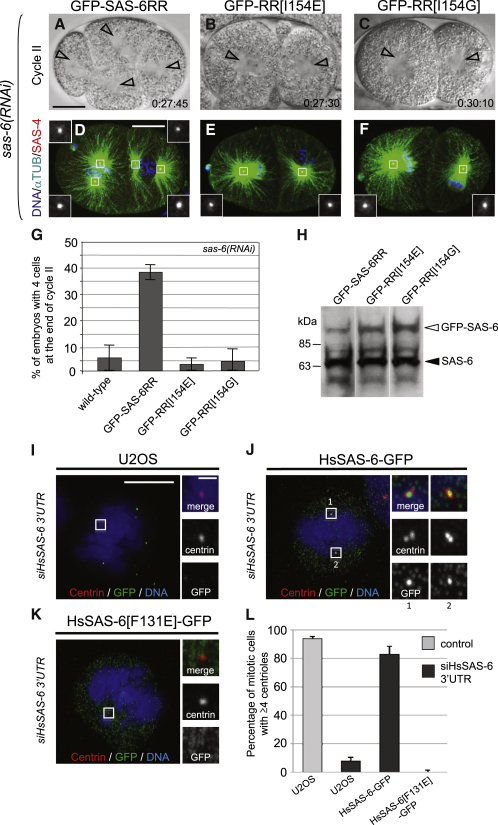
Functional Analysis of SAS-6 in *C. elegans* and Human Cells (A)–(F) Anterior is to the left and scale bar is 10 μm. (A–C) Images at the end of the second cell cycle from representative DIC recordings of embryos treated with *sas-6*(*RNAi*) and expressing GFP-SAS-6RR (A), GFP-SAS-6RR[I154E] (B), or GFP-SAS-6RR[I154G] (C). Elapsed time after pronuclear meeting is indicated in minutes and seconds; arrowheads indicate centrosomes. (D–F) Embryos during mitosis of the second cell cycle treated with *sas-6*(*RNAi*) and expressing GFP-SAS-6RR (D), GFP-SAS-6RR[I154E] (E), or GFP-SAS-6RR[I154G] (F) stained with antibodies against α-tubulin (green) and SAS-4 (red); DNA in blue. Insets show an ∼2.5-fold magnified view of one MTOC. Note that GFP-SAS-6RR[I154E] and GFP-SAS-6RR[I154G] are not present at centrioles (data not shown), presumably because they fail to be incorporated as a result from the lack of oligomerization. (G) Quantification of experiments illustrated in (A)–(C). The percentages of embryos with four cells at the end of the second cell cycle are indicated (n = 31 for wild-type, n = 37 for GFP-SAS-6RR, n = 50 for GFP-SAS-6RR[I154E], and n = 35 for GFP-SAS-6RR[I154G]). Shown are the mean percentages ± SEM from two independent experiments. (H) Western blot analysis of GFP-SAS-6RR, GFP-SAS-6[I154E], or GFP-SAS-6RR[I154G] embryonic extracts probed with SAS-6 antibodies to reveal both endogenous protein (filled arrowhead) and GFP fusions (open arrowhead). (I–K) Metaphase U2OS, iU2OS:HsSAS-6-GFP, and iU2OS:HsSAS-6[F131E]-GFP cells transfected with siRNAs targeting the 3′UTR of endogenous HsSAS-6 (siHsSAS-6-3′UTR), induced concomitantly with doxycycline, fixed after 48 hr, and stained with antibodies against centrin (red) and GFP (green); DNA in blue. Scale bar, 10 μm. Insets show magnified view of the delineated regions; scale bar in insets, 1 μm. Whereas the vast majority of mitotic cells expressing HsSAS-6[F131E]-GFP did not exhibit centriolar GFP (see C), a centriolar signal was detected earlier during the cell cycle in most cells (data not shown), suggestive of a failure in stable incorporation as a result of the lack of oligomerization. (L) Percentage of cells in mitosis (prophase to metaphase) with four or more centrioles after 48 hr treatment with Stealth RNAi Low GC negative control or siHsSAS-6-3′UTR (n = 135 for U2OS + control siRNA, n = 236 for U2OS + 3′UTR siRNA, n = 226 for U2OS + 3′UTR siRNA + HsSAS-6-GFP, and n = 160 for U2OS + 3′UTR siRNA + HsSAS-6[F131E]-GFP). Data from at least three independent experiments (≥50 cells/experiment) are shown; error bar indicates SEM. See also Figure S3, Movies S1, Movie S2, and Movie S3.

To investigate the biological significance of the oligomerization of SAS-6 homodimers mediated by the N-N interaction, we generated transgenic worms expressing GFP fused to SAS-6[I154E] engineered so as to be resistant to RNAi directed against endogenous SAS-6 (GFP-SAS-6RR[I154E]) (Dammermann et al., 2008). A similar approach was utilized to replace I154 by glycine, a smaller and noncharged residue, thus generating GFP-SAS-6RR[I154G]. Upon *sas-6*(*RNAi*) in an otherwise wild-type background, the two paternally contributed centrioles split from one another and assembled a bipolar spindle at the end of the one-cell stage (Movie S1). In contrast, a monopolar spindle usually assembled in each blastomere at the end of the second cell cycle (Figure 3G). In *sas-6*(*RNAi*) embryos expressing RNAi-resistant wild-type SAS-6 fused to GFP (GFP-SAS-6RR), ∼40% of embryos underwent bipolar spindle assembly in each blastomere at the end of the second cell cycle (Figures 3A and 3G and Movie S2) (Kitagawa et al., 2009). This reflected rescue of centriole formation, as demonstrated by the presence of the centriolar protein SAS-4 in each spindle pole (Figure 3D). Partial rescue to only ∼40% is likely due to GFP at the N terminus interfering with the function of SAS-6 and to levels of the fusion protein being lower than that of the endogenous protein (Figure 3H) (see also Kitagawa et al., 2009). Importantly, there was no rescue of centriole formation in *sas-6*(*RNAi*) embryos expressing GFP-SAS-6RR[I154E] or GFP-SAS-6RR[I154G] (Figures 3B, 3C, and 3E–3G and Movie S3). This was not due to differences in expression levels; in fact, GFP-SAS-6RR[I154E] and GFP-SAS-6RR[I154G] were expressed at slightly higher levels than wild-type GFP-SAS-6RR (Figure 3H). We conclude that I154 is essential for centriole formation in *C. elegans*.

We then addressed whether the importance of oligomerization mediated by the N-N interaction is evolutionarily conserved. To this end, we analyzed the human protein HsSAS-6, in which the residue corresponding to *C. elegans* I154 is F131 (Figure S2). We generated constructs in which wild-type or F131E mutant HsSAS-6 was fused to GFP and expressed from a doxycycline-inducible promoter (Bach et al., 2007). The fusion constructs do not contain the 3′UTR of the endogenous gene, so we targeted this region using siRNAs to deplete solely endogenous HsSAS-6 without affecting the GFP fusion proteins (Figure S3E). Whereas ∼95% of control mitotic cells harbored the usual number of ≥ 4 centrioles marked by the EF-hand protein centrin (Figure 3L), this was the case for only ∼10% of mitotic cells treated with siHsSAS-6-3′UTR (Figures 3I and 3L). Expression of wild-type HsSAS-6-GFP in cells treated with siHsSAS-6-3′UTR resulted in substantial rescue of centriole formation, with > 80% of mitotic cells harboring ≥ 4 centrioles (Figures 3J and 3L). By contrast, cells expressing HsSAS-6[F131E]-GFP and subjected to siHsSAS-6-3′UTR did not exhibit rescue (Figures 3K and 3L).

Together, these experiments demonstrate that a singly conserved residue mediating the SAS-6 N-N interaction is essential for centriole formation in *C. elegans* and in human cells, indicating that the capacity to oligomerize is critical for the function of SAS-6 proteins across evolution.

### Root of the 9-Fold Symmetry of Centrioles

**Figure 4:**
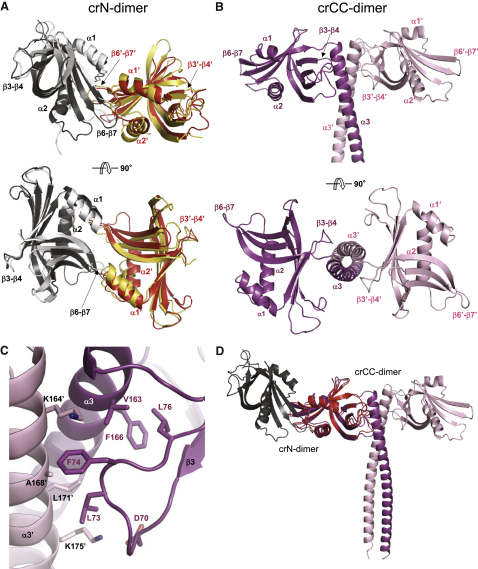
Structural Analysis of *C. reinhardtii* Bld12p (A) Two overall views of the crN-dimer structure 90° apart and superimposed onto the ceN-dimer structure. Monomers A and B are depicted in cartoon representations and colored in dark gray and red (crN-dimer) and light gray and yellow (ce-N dimer), respectively. The global superimposition yielded a root-mean-square deviation of 1.6 Å for 217 backbone atoms. (B) Two overall views of the crCC-dimer structure 90° apart. Monomers A and B are colored in magenta and light pink, respectively. (C) Close-up views of the interaction network seen at the crCC-dimer interface in cartoon (main chains) and stick (contacting residues) representations. Key secondary structure elements are assigned. Oxygen and nitrogen atoms are colored in red and blue, respectively. Carbon atoms are colored in magenta and light pink. (D) Superimposition of monomer B of the crN-dimer onto monomer A of the crCC-dimer. The resulting assembly was used as a template for building the Bld12p ring structure shown in Figure 5. See also Figure S2 and Figure S4.

**Figure S4:**
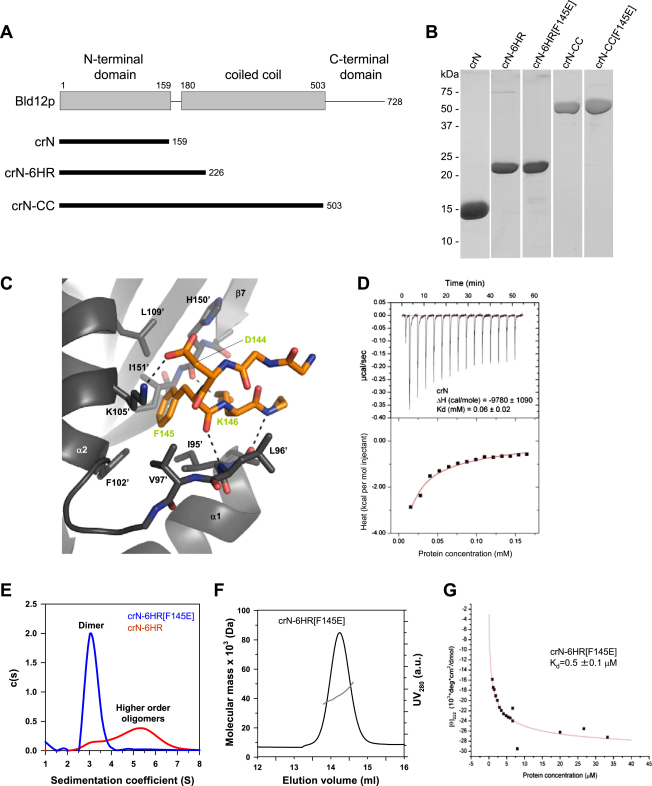
Characterization of *C. reinhardtii* Bld12p, Related to Figure 4 (A) Schematic representation of Bld12p and fragments generated in this study. crN, N-terminal domain; crN-6HR, N-terminal domains extended by 6 heptad repeats of the adjacent coiled coil; crN-CC; N-terminal domains extended by the full adjacent coiled coil. Numbers correspond to Bld12p amino acids. (B) Coomassie-stained SDS-PAGE sections showing final purification products of the indicated recombinant proteins. Approximate molecular weights from in-gel markers are shown. (C) Close up view of the interaction network seen at the crN-dimer interface in cartoon (main chains) and stick (contacting residues) representations. Monomers A and B are colored in dark gray and orange, respectively. (D) Dissociation isotherm obtained by ITC for crN. Top panel: raw data representing the response to injections of crN at high concentration into sample buffer. Bottom panel: integrated heat change (closed squares) and associated curve fit (red solid line). (E) Sedimentation velocity analysis of the crN-6HR (red) and crN-6HR[F145E] (blue). Protein concentration was 150 μM for both samples. The peak labeled with ‘Dimer’ corresponds to a molecular weight of ∼50 kDa, which is consistent with the formation of dimers. The region of S values highlighted with ‘Higher order oligomers’ is indicative of higher order oligomer formation beyond dimers. (F) MALS analysis of crN-6HR[F145E]. The UV absorbance profile of size exclusion chromatography (black line) is overlaid with the molecular weight estimation by multi-angle light scattering (gray line). The determined molecular weight of 48.3 kDa is consistent with the formation of a stable dimer. Molecular weight of the crN-6HR[F145E] monomer: 25.8 kDa. (G) crN-6HR[F145E] dilution series monitored by CD at 222 nm. The red solid line represents the fit to the data (closed squares) using a monomer-dimer model.

To understand the function of SAS-6 oligomerization for centriole formation at the structural level, we investigated the molecular properties of a SAS-6 protein from an organism in which the cartwheel has a canonical structure. This is the case in human cells (Guichard et al., 2010), but recombinant HsSAS-6 and fragments thereof were not soluble (data not shown). By contrast, we were able to produce soluble recombinant proteins from *C. reinhardtii* Bld12p (Nakazawa et al., 2007), which has the same domain organization as other SAS-6 orthologues (Figure S4A). Like for *C. elegans* SAS-6, we started by producing a fragment encompassing the N-terminal domain (denoted crN; Figures S4A and S4B) and solved its structure to 2.1 Å resolution by X-ray crystallography (Table S1). We found that the asymmetric unit of the crystal contained three equivalent crN dimers (denoted the crN-dimer hereafter). The monomers in each dimer are related by local 2-fold symmetry (Figure 4A). The overall structure and organization of the crN-dimer, as well as the F145 residue engaged at the N-N interface and corresponding to I154 of *C. elegans* SAS-6, are similar to that of the *C. elegans* ceN-dimer (Figure 4A and Figure S4C). The stability of the crN-dimer in solution was assessed by ITC, and the K_d_ was determined to be 60 ± 20 μM (Figure S4D), which is similar to that of the ceN-dimer from *C. elegans* (see Figure 2E). Overall, these results indicate that there is strong structural conservation among N-terminal domains of SAS-6 proteins across evolution. In addition, they demonstrate that the function of the critical residue within the β6-β7 loop mediating the interaction between pairs of N-terminal domains is likewise conserved.

To investigate the structural organization of the Bld12p N-terminal domains in the context of the two-stranded parallel coiled coil, we produced a fragment in which the crN variant was extended by the first six heptad repeats of the Bld12p coiled coil (crN-6HR; Figures S4A and S4B). AUC experiments conducted at 150 μM protein concentration revealed that crN-6HR forms higher-order oligomeric species (Figure S4E). However, a mutant in which F145 was substituted for glutamate (crN-6HR[F145E]) formed only dimers, as revealed by AUC and MALS experiments (Figures S4E and S4F). The stability of crN-6HR[F145E] was assessed by CD, which yielded a K_d_ of 0.5 ± 0.1 μM (Figure S4G). We solved the structure of Bld12p crN-6HR[F145E] to 3.0 Å resolution by X-ray crystallography. The asymmetric unit of the crystal revealed a dimer (denoted the crCC-dimer hereafter; Figure 4B). Dimerization is brought about by interactions between the two α3 helices, which establish a parallel, two-stranded coiled coil through knobs-into-hole packing of the residues occupying the heptad a and d core positions. The relative orientation of the two N-terminal domains is maintained by predominantly hydrophobic interactions formed between residues of their β3-β4 loops and residues from both coiled-coil α3 helices (Figure 4C).

**Figure 5:**
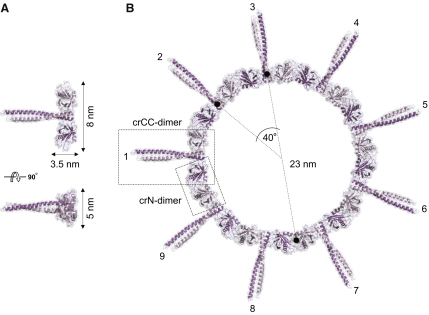
Structural Model of *C. reinhardtii* Bld12p Ring Oligomer (A) Two views of the crCC-dimer building block 90° apart. (B) Nine crCC-dimers were associated such that their N-terminal domains interact as observed in the crN-dimer (Figure 4D). The resulting 9-fold symmetric ring oligomer exhibits a diameter of ∼23 nm and a thickness of ∼3.5 × 5 nm. The long axis of the coiled-coil domains are in plane with and radiate out from the ring. See also Figure S5.

**Figure S5:**
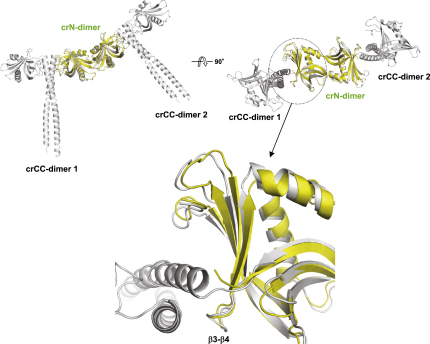
Modeling of *C. reinhardtii* Bld12p Oligomers, Related to Figure 5 Only small differences are observed between crN-dimers (yellow, chains C and D are shown) and the corresponding dimer resulting from modeling of the idealized 9-fold symmetric ring using crCC-dimers (light gray). Note the close fit in the position of the β3-β4 loop (close-up), which is critically involved in determining the CC-N interface.

Having the structures of both the crN-dimer and the crCC-dimer of Bld12p allowed us to build a structural model of higher-order oligomers using both dimer interfaces. Strikingly, when crCC-dimers were associated such that their N-terminal domains interact as observed in the crN-dimer (Figure 4D and Figure S5), we obtained a ring with a 9-fold symmetry (Figure 5; see Experimental Procedures for full description of the modeling). In this structural model, the long axes of the coiled-coil domains are in plane with and radiate out from the ring, which is ∼3.5 × 5 nm in thickness and ∼23 nm in mean diameter (Figure 5B).

**Figure 6:**
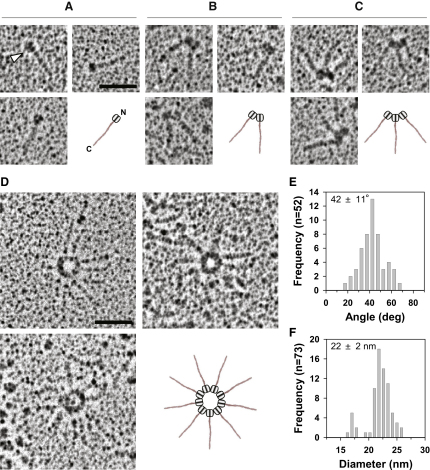
Electron Microscopy of *C. reinhardtii* Bld12p (A–D) Rotary metal shadowing electron micrographs of crN-CC specimens. Schematic interpretations of the specimens are indicated. Note that not all nine spokes represented in the schematic of (D) are unambiguously discerned in the electron micrographs, presumably because some of them are perturbed during sample preparation. The arrow in (A) highlights the head-like moieties of crN-CC. Scale bars, 50 nm. (E) Histogram representation of angles measured between the two legs of the V-shaped crN-CC specimens shown in (B) (n = 51). (F) Histogram representation of mean diameters measured from crN-CC ring oligomers shown in (D) (n = 73). The majority of rings possess a diameter of 22 nm, which is in good agreement with the 23 nm diameter determined from the 9-fold symmetric structural ring model of Bld12p (Figure 5). Note also that a minor fraction of crN-CC rings displayed a lower than 22 nm diameter, which probably correlates with a different number of crN-CC dimers. See also Figure S6.

A key prediction of our structural model is that Bld12p possesses properties to self-assemble into a ring with 9-fold symmetry. We tested this hypothesis by performing electron microscopy experiments with bacterially expressed Bld12p. As the full-length protein exhibited unspecific aggregation (data not shown) and as the C-terminal part of Bld12p is not evolutionarily conserved and is predicted to be largely disordered, we produced a Bld12p fragment encompassing the N-terminal and coiled-coil domains (crN-CC; Figures S4A and S4B). Electron microscopy revealed that crN-CC is an elongated ∼40 nm rod that displays a globular head-like moiety at one extremity (Figure 6A). The overall organization of crN-CC is similar to that of the *C. elegans* SAS-6 homodimer, with the rod corresponding to the two-stranded parallel coiled coil and the head moiety to the two N-terminal domains (compare Figure 6A with Figure 1B). Strikingly, at increased concentrations, crN-CC could associate in a head-to-head fashion to form an overall V-shaped structure (Figure 6B). The angle between the two legs of the V was determined to be 42 ± 11° (Figure 6E), which suggestively corresponds to approximately one-ninth of 360°.

**Figure S6:**
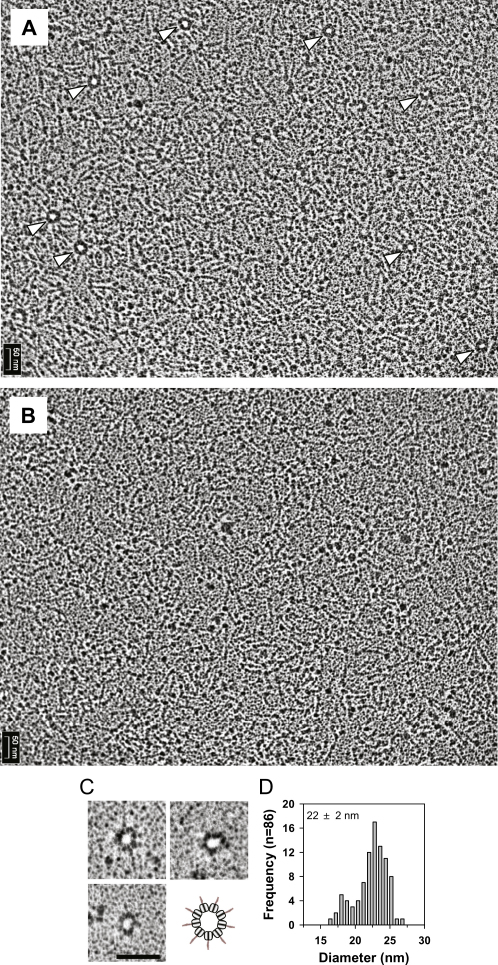
Electron Microscopy of *C. reinhardtii* crN-CC and crN-6HR, Related to Figure 6 (A, B) Electron micrographs of crN-CC (A) and crN-CC[F145E] (B) after glycerol spraying and rotary metal shadowing. Protein concentration was 1 mg/ml for both samples. Scale bars are indicated. Arrows in panel (A) highlight the ring oligomers only obtained with crN-CC. (C) Electron micrographs of crN-6HR ring oligomers after glycerol spraying and rotary metal shadowing. Scale bar, 50 nm. (D) Histogram representation of mean diameters measured from crN-6HR ring oligomers shown in (C). Note that the observed mean ring diameter and their distribution are very similar to those observed with crN-CC (Figure 6F).

Remarkably, we found in addition that crN-CC further assembled into higher-order oligomers (Figure 6C) and could form ring-like structures from which emanated spokes corresponding to the coiled-coil domains (Figure 6D and Figure S6A). The mean diameter of the central ring was 22 ± 2 nm (Figure 6F), which is similar to that of the crCC-dimer ring model (Figure 5B) and of the central hub of the *C. reinhardtii* cartwheel (Cavalier-Smith, 1974). In contrast, no higher-order assemblies were obtained with an crN-CC mutant in which glutamate was substituted for F145 (crN-CC[F145E]; Figure S6B), demonstrating that this residue is critical for forming V-shaped structures and ring oligomers. Consistent with the findings with crN-CC, the shorter crN-6HR fragment also formed predominantly rings with a diameter similar to that of crN-CC, although radial spokes were not observed in this case given the small size of the crN-6HR coiled coil (Figures S6C and S6D). Collectively, these data demonstrate that Bld12p self-assembles into ring-like structures from which emanate radial spokes.

## Discussion

**Figure 7:**
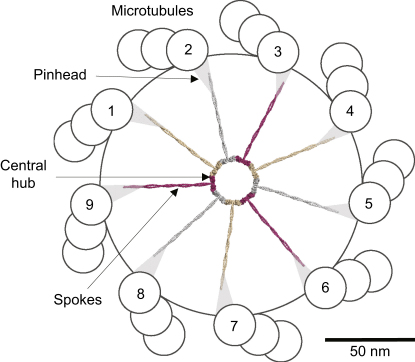
Structural Model of the SAS-6-Based Cartwheel in the Context of the Centriole In this model, viewed from the proximal end, the SAS-6 coiled-coil domains would contribute substantially to the formation of the spokes of the cartwheel and possibly connect to the pinheads. The coiled-coil domains were extended to the size expected from structure prediction, and their lengths fit well with the ∼40 nm length measured from electron micrographs of crN-CC specimens (Figure 6A). However, we note that the protein Bld10p from *C. reinhardtii*, which localizes to the pinhead of the cartwheel, also contributes to the formation of the spokes (Hiraki et al., 2007). Scale bar, 50 nm.

The 9-fold symmetry of centrioles, cilia, and flagella has fascinated biologists since it was discovered decades ago with the advent of electron microscopy. The mechanisms at the origin of this remarkable 9-fold symmetry have inspired many hypotheses (reviewed in Strnad and Gönczy, 2008). For instance, because duplication of the centriole occurs once per cell cycle, as is the case for replication of the genetic material, it has been proposed that centriole formation may similarly rely on nucleic acids (reviewed in Marshall and Rosenbaum, 2000). Our work demonstrates that a protein-based mechanism is sufficient to account for an initial step of centriole formation, as the self-assembly properties of SAS-6 generate a molecular architecture with a 9-fold symmetry that bears striking resemblance with the cartwheel. The cartwheel has been perhaps best described in *C. reinhardtii* and consists of a central hub from which emanate nine spokes capped by a pinhead-like structure (Figure 7) (Cavalier-Smith, 1974). The cartwheel is the first structure with a 9-fold symmetry apparent at the onset of centriole formation, which has led to the suggestion that it acts as a scaffold onto which centriolar microtubules then assemble (reviewed in Strnad and Gönczy, 2008). Support for this view has come notably from the analysis of *bld12* mutants in which the cartwheel is missing (Nakazawa et al., 2007). In most cells null for *bld12* function, basal bodies are fragmented into pieces, indicating that the cartwheel is required for centriole formation. Interestingly, in addition, the rare mutant cells that harbor basal bodies exhibit defects in the 9-fold symmetry, with the number of microtubule blades varying from 7 to 11. This observation strongly supports the notion that the cartwheel is critical for dictating the 9-fold symmetry of centrioles.

Our findings elucidate the structural basis of the cartwheel and thus of the 9-fold symmetry of centrioles. We first establish that proteins of the SAS-6 family form coiled-coil-mediated homodimers. Our elongated molecular model of SAS-6 and Bld12p homodimers is in contrast to the proposal that *Drosophila* DmSas6 exhibits a globular arrangement (Gopalakrishnan et al., 2010). Although this may reflect a *Drosophila*-specific feature, we note that all proteins of the SAS-6 family contain a predicted coiled-coil domain that is expected to form an extended rod (Carvalho-Santos et al., 2010). Our work further reveals that interaction between homodimers mediated by adjacent N-terminal domains results in the oligomerization of SAS-6 homodimers. Strikingly, in addition, recombinant Bld12p homodimeric building blocks self-assemble into an ∼22 nm ring from which the coiled-coil domains emanate radially. The overall appearance of Bld12p oligomers in our electron micrographs, as well as that of our structural ring model, is remarkably similar to the cartwheel comprising a central hub and radial spokes, as observed in vivo (Figure 7) (Cavalier-Smith, 1974).

Although new centrioles form next to the old ones in most proliferating cells, once per cell cycle, centrioles can also assemble de novo, for instance in multiciliated epithelial cells or after ablation of the resident centrioles (Khodjakov et al., 2002; Loncarek and Khodjakov, 2009; Dirksen, 1991). Moreover, the cartwheel also possesses self-assembling properties (Gavin, 1984). Our findings provide an attractive mechanism for how de novo centriole formation may be achieved, as self-assembly of SAS-6 proteins is sufficient to mediate formation of a structure that bears resemblance to the cartwheel. Although centrioles can assemble de novo in some cases, they form strictly in the vicinity of the old centriole in most proliferating cells. We speculate that this may reflect the fact that the vicinity of the old centriole is a favorable environment for promoting self-assembly of SAS-6 proteins, perhaps because of the local enrichment of other centrosomal components. Alternatively, phosphorylation of SAS-6, for instance as is known to occur in *C. elegans* through the action of the kinase ZYG-1 (Kitagawa et al., 2009), could regulate the formation or stability of SAS-6 oligomers. Regardless, it will be of utmost interest to elucidate how the basic ring of SAS-6 homodimers is stabilized so that it can promote the formation of a mature centriole.

In light of the importance of regulated centrosome duplication in genome stability (reviewed in Nigg and Raff, 2009), the structural information uncovered in this study, and in particular the identification of the residues mediating interaction between adjacent SAS-6 N-terminal domains, represents a promising avenue to modulate centriole formation for therapeutic purposes. Furthermore and in conclusion, because these residues are well conserved among SAS-6 orthologs, we propose that the self-assembly of SAS-6 homodimers into a 9-fold symmetric ring structure is a fundamental property at the root of the universal 9-fold symmetry of centrioles.

## Experimental Procedures

### Protein Preparation and Biophysical Characterization

Standard cloning and recombinant protein production in bacteria is described in the Supplemental Information. Protein identity was confirmed by ESI-TOF mass spectrometry and concentrations estimated by UV at 280 nm.

CD spectra were collected at 10°C at a protein concentration of 25 μM in 20 mM Na_2_HPO_4_, 150 mM NaCl (pH 7.4) (PBS) using a Chirascan spectropolarimeter (AppliedPhotophysics) with a 0.1 cm path length. Thermal stability experiments were performed using a 1°C/min temperature ramp between 10°C and 90°C and monitored by CD at 222 nm. The dissociation constant of ceCC and crN-6HR[F145E] was determined by monitoring the CD signal at 222 nm and at 20°C after buffer signal subtraction in a dilution series. The samples were reduced with DTT prior to data acquisition to ensure that no covalent dimers remained. The concentration-dependent mean residue elipticity at 222 nm was fit to a two-state association model to obtain the K_d_.

MALS was performed in PBS supplemented with 1 mM DTT using an S-200 analytical size exclusion chromatography column connected in-line to miniDAWN TREOS light scattering and Optilab T-rEX refractive index detectors (Wyatt Technology). Samples of 2–4 mg/ml concentration were used.

AUC experiments were performed at 20°C using 0.15–0.3 mM proteins in 20 mM Tris-HCl (pH 7.5), 150 mM NaCl, 2 mM TCEP, and 1% glycerol using a ProteomeLab XL-I analytical ultracentrifuge (Beckman). All sedimentation velocities were recorded by measuring absorbance at 280 or 290 nm, with 200 scans every 4 min at 35000 rpm. Data were processed using SEDFIT (Schuck, 2000). Partial specific volume was calculated from the amino acid sequence.

ITC experiments were performed at 7°C using an ITC200 system (Microcal). 1.0–1.6 mM samples of ceN and crN in 20 mM sodium phosphate (pH 7) supplemented with 100 mM NaCl and 1 mM DTT (ceN) or 12 mM HEPES (pH 7) supplemented with 100 mM NaCl and 0.7 mM βMe (crN) were loaded for stepwise injection into sample buffer alone. The resulting heats were integrated using Origin (OriginLab) and fit with the two-step dissociation model provided by the software package.

Cysteine crosslinking of SAS-6 ceCC was performed using protein samples of 20 μM concentration in PBS buffer without DTT. Substantial crosslinked dimer formation was observed on nonreducing SDS-PAGE after overnight incubation at 20°C.

### Electron Microscopy

Electron micrographs were taken in a Philips Morgagni TEM operated at 80 kV equipped with a Megaview III CCD camera. Protein samples (0.1–1 mg/ml) in PBS were supplemented with glycerol to a final concentration of 30%. Samples were subsequently sprayed onto freshly cleaved mica and rotary shadowed in a BA 511 M freeze-etch apparatus (Balzers) with platinum/carbon at an elevation angle of 3°–5° (Fowler and Aebi, 1983). Mean diameters of individual crN-CC and crN-6HR specimens were determined by taking the arithmetic middle of the outer and inner diameter of the ring specimens.

### Structure Determination

Structure solution by X-ray crystallography is described in full in the Supplemental Information. In brief, crystals of the *C. elegans* ceN fragment in which Ser123 was mutated to glutamate (ceN[S123E]) (Kitagawa et al., 2009) diffracted to 2.1 Å resolution. Phase information was obtained by SAD using NdCl_3_ derivatized crystals and the structure refined to final *R*_work_/*R*_free_ values of 21.0%/25.7%.

Crystals of the *C. reinhardtii* crN and crN-6HR[F145E] fragments diffracted to 2.1 and 3.0 Å resolution, respectively. The structures of both proteins were solved by molecular replacement and refined to final *R*_work_/*R*_free_ values of 18.1%/21.8% (crN) and 19.6%/22.9% (crN-6HR[F145E]). See Table S1 for data collection and refinement statistics.

X-ray data were collected at beamlines X06DA and X06SA of the Swiss Light Source (Paul Scherrer Institut, Villigen, Switzerland).

### Modeling

Structure determination of the crCC-dimer and the crN-dimer of *C. reinhardtii* Bld12p revealed two distinct types of interfaces. Analysis of the three crN-dimers (denoted AB, CD, and EF) within the asymmetric unit of the crystal revealed small differences between them (rmsd values of 0.5–1.0 Å). crCC-dimers were continuously associated such that their N-terminal domains interact as observed for the AB, CD, or EF crN-dimers, resulting in flat left-handed helices with 10–11 dimers per turn, with diameters of ∼23–27 nm and pitches of ∼80–165 Å. In these assemblies, the coiled coils radiate out from and are nearly perpendicular to the helix axis. To assist modeling of a 9-fold symmetric ring, a planar wheel with spokes every 40° was generated, and a Cα model of the crCC-dimer structure was positioned with its 2-fold axis aligned with one spoke. Radial position (along the spoke) and orientation (rotation around the spoke axis) were optimized such that the resulting N-N interaction with a neighboring crCC-dimer generated by a 40° rotation became as close as possible to that observed for crN-dimers. The fit was assessed by comparing the generated N-N interaction with the structures of the AB, CD, and EF crN-dimers. After optimization, superposition of the generated “40°-model” with the AB, CD, or EF crN-dimers yielded rmsd values of 1.3 Å, 1.7 Å, and 1.3 Å, respectively. Figure S5B shows the superposition of the optimized 40°-model with the CD crN-dimer.

The small differences between the N-N contact generated as described above in the model and that observed in the crystal structure makes the existence of a ring very plausible. In reality, structural adjustments are expected to be distributed more globally and over many degrees of freedom and to not be locally concentrated in the interface between pairs of N-terminal domains as in the simplified modeling approach. In particular, small changes in the coiled coil as well as in the coiled coil-N-terminal domain interfaces would also be expected.

### Nematode Strains and RNA Interference

For the experiments with the RNAi-resistant strains, GFP-SAS-6RR (Dammermann et al., 2008) and all other strains were maintained according to standard procedures. For generating GFP-SAS-6RR[I154E] and GFP-SAS-6RR[I154G] transgenic lines, appropriate primers (sequences available upon request) were used to PCR-amplify *sas-6* cDNA, replacing the ATT that normally codes I154 by GAA or GGA, respectively, and cloning the resulting fragments into pIC26, a *pie-1*-based vector containing a rescuing *unc-119* cDNA (gift from Karen Oegema). Sequence-verified plasmids were bombarded, yielding two integrated strains for both strains.

RNAi-mediated inactivation was performed by soaking (Maeda et al., 2001). In brief, L4 larvae were placed in a solution containing in vitro synthesized dsRNAs targeting a portion of *sas-6* corresponding to the engineered RNAi-resistant construct (Dammermann et al., 2008), incubated for 24 hr at 20°C, and allowed to recover for 12 hr at 20°C before analysis.

Cell-cycle progression in *C. elegans* early-stage embryos was monitored by time-lapse differential interference contrast (DIC) microscopy, recording one image every 5 s at 23°C.

### Indirect Immunofluorescence and Western Blot Analysis for *C. elegans*

Embryos were fixed and stained essentially as described (Leidel et al., 2005). In brief, embryos were methanol fixed for < 3 min and blocked in 3% bovine serum albumin (BSA) for > 20 min prior to incubation with primary antibodies overnight at 4°C. Primary antibodies were 1:800 SAS-4 (rabbit) (Leidel and Gönczy, 2003) and 1:200 α-tubulin (mouse, DM1α, Sigma). Secondary antibodies were goat anti-mouse coupled to Alexa 488 and goat anti-rabbit coupled to Alexa 568 (Molecular Probes), both used at 1:500. Slides were counterstained with ∼1 μg/ml Hoechst 33258 (Sigma) to reveal DNA.

Indirect immunofluorescence was imaged on a Leica SP2 confocal microscope. Optical sections were acquired every 0.25–0.3 μm, and planes containing centrioles were projected together. A similar procedure was applied for microtubules and DNA. Images were processed using ImageJ and Adobe Photoshop, preserving relative image intensities within a series.

For western blot analysis, transgenic worms expressing GFP-SAS-6RR, GFP-SAS-6RR[I154E], or GFP-SAS-6RR[I154G] were collected in Laemmli SDS sample buffer, boiled, and subjected to SDS-PAGE, and signal intensities were analyzed after western blotting using 1:200 SAS-6 antibody (Leidel et al., 2005). HRP-conjugated anti-rabbit antibodies (Amersham) were utilized as secondary at 1:5000. The signal was detected with chemiluminescence (Roche or Pierce).

### Cell Culture and Transfections

U2OS cells were obtained from the EACC and maintained in McCoy's 5A GlutaMAX medium (Invitrogen) supplemented with 10% fetal bovine serum (FBS) for U2OS cells or tetracycline-negative FBS (Brunschwig) for the inducible episomal cell lines (iU2OS). To generate such iU2OS lines, U2OS cells were transfected with pEBTet-HsSAS-6-GFP or pEBTet-HsSAS-6[F131E]-GFP using Lipofectamine2000 (Invitrogen). Transfected cells were selected with 1 μg/ml puromycin 1 day after transfection and amplified. Early passage cells were used, inducing expression with 1ug/ml doxycycline for 48 hr.

Endogenous HsSAS-6 was depleted using a Stealth RNAi siRNA (Invitrogen) targeting the 3′UTR of HsSAS-6 (5-GAGCUGUUAAAGACUGGAUACUUUA-3). Stealth RNAi siRNA negative control LO GC (Invitrogen) was used as a control.

siRNA transfection was performed using Lipofectamine RNAiMax (Invitrogen) according to the manufacturer's protocol, and cells were analyzed 48 hr after siRNA treatment.

### Cell-Extract Preparation and Biochemical Assays

Cells were collected, washed in PBS, and lysed on ice for 30 min in lysis buffer (15 mM Tris-HCl [pH 7.5], 150 mM NaCl, 2.5 mM MgCl_2_, 0.5% NP-40, 50 mM NaF, and 0.2 mM orthovanadate; Complete Mini Protease Inhibitor Cocktail [Roche Diagnostics]). Lysates were cleared by centrifugation for 15 min at 13,000 × g at 4°C and the supernatant collected. SDS-PAGE was performed using 4%–15% polyacrylamide gradient gels (BioRad), followed by transfer on nitrocellulose membrane (Amersham). The membrane was probed with mouse HsSAS-6 antibody (Santa Cruz, 1:1000) or rabbit Actin antibody (Abcam, 1:2000), followed by incubation with their respective HRP-conjugated secondary (Promega) and the signal detected with chemiluminescence.

### Immunofluorescence and Microscopy for Human Cells

U2OS cells grown on glass coverslips were fixed for 7–10 min in –20°C methanol, washed in PBS, and blocked in 1% bovine serum albumin and 0.05% Triton X-100 in PBS. Cells were incubated 2 hr at room temperature or overnight at 4°C with primary antibodies, washed three times for 5 min in PBST (0.05% Triton X-100 in PBS), incubated 45 min at room temperature with secondary antibodies, stained with ∼1 μg/ml Hoechst 33258, washed three times in PBST, and mounted. Primary antibodies were 1:4000 mouse centrin (20H5; gift from Jeffrey L. Salisbury) and 1:500 rabbit GFP (gift from Viesturs Simanis). Secondary antibodies were 1:1000 goat anti-rabbit coupled to Alexa 488 and 1:1000 goat anti-mouse coupled to Alexa 568. For quantification of centrioles, mitotic cells (prophase to metaphase) with similar cytoplasmic GFP expression were used; highly expressing cells that often harbored GFP aggregates were not retained for analysis. Imaging was done on a Zeiss LSM710 confocal microscope. Optical sections were acquired every 0.12 μm, and planes containing centrioles were projected together. Images were processed using ImageJ and Adobe Photoshop, preserving relative image intensities within a series.

**Extended Experimental Procedures**

**Cloning and Protein Preparation**

**C. elegans *SAS-6 Proteins***

DNA encoding full-length or fragments of *C. elegans* SAS-6 (Uniprot ID O62479) were cloned in pET system vectors (Novagen) encoding for N-terminal His_6_-tags or pGEX system vectors (GE healthcare) encoding for N-terminal GST-tags. Full-length SAS-6 and the ceN-CC fragment (residues 1-414) were cloned in pET30a, the ceCC fragment (residues 181-408) in pET15b and the ceN fragment (residues 1-168) in a modified pET15b vector. For full-length SAS-6, the ceN fragment and ceN-CC for electron microscopy, recombinant protein expression was performed in *Escherichia coli* strain BL21 gold (DE3) in Luria-Bertani (LB) medium. Protein expression was induced at 23°C by addition of 0.4 mM IPTG and allowed to proceed for 16h. Cell pellets were lysed by lysozyme treatment and sonication, resuspended in lysis buffer containing 50 mM Tris-HCl (pH 7.5), 500 mM NaCl, 20 mM imidazole and 0.5% Triton X-100. The lysates were incubated with Ni-NTA agarose beads (QIAGEN). The beads were then washed ten times with lysis buffer. Proteins were eluted from the beads with a buffer containing 80 mM PIPES-KOH (pH 6.8), 80 mM KCl, 2 mM MgCl_2_ and 400 mM imidazole, followed by size exclusion chromatography in 10 mM HEPES (pH 7.2), supplemented with 150 mM NaCl and 5 mM DTT. His_6_-tags were removed by thrombin prior to the final purification step.

For the ceCC fragment, cell pellets were resuspended in PBS (20 mM Na_2_HPO_4_, 150 mM NaCl, pH 7.4) supplemented with 8 M Urea, and cells lysed by repeated cycles of freeze-thawing. Protein was purified from cell lysate supernatants by metal affinity chromatography as described above but in the presence of 8 M Urea, and dialyzed against thrombin cleavage buffer. Removal of the His_6_-tags by thrombin was followed by size exclusion chromatography in PBS supplemented with 0.5 mM DTT.

For the AUC experiments, the recombinant protein expression of ceN-CC was performed as described above. Cell pellets were lysed by lysozyme treatment and sonication, resuspended in lysis buffer containing 50 mM Tris-HCl (pH 7.5), 500 mM NaCl, 5 mM EDTA, 1 mM DTT, 1:1000 protease inhibitor cocktail (Sigma) and 0.5% Triton X-100. The lysates were incubated with Glutathion sepharose beads (GE healthcare). The beads were then washed ten times with lysis buffer supplemented with additional 500 mM NaCl. Proteins were eluted from the beads by removal of the GST-tags by prescission protease (GE healthcare) in a buffer containing 20 mM Tris-HCl, pH 7.5, 150 mM NaCl, 0.5 mM EDTA, 1 mM DTT, followed by dialysis in 20 mM Tris-HCl, pH 7.5 supplemented with 150 mM NaCl and 2 mM TCEP. Proteins were concentrated by centrifugal ultrafiltration.

**C. reinhardtii *Bld12p Proteins***

The DNA fragments coding for crN-CC (residues 1-503) and crN-CC[F145E], crN (residues 1-159), and crN-6HR (residues 1-226) and the crN-6HR[F145E] were PCR-amplified from a full-length *C. reinhardtii* Bld12p clone (kind gift by Masafumi Hirono; Uniprot ID A9CQL4). Cloning of PCR fragments into the pET-based bacterial expression vector PSTCm1 (crN and crN-6HR[F145E]) was performed using a Positive Selection method (Olieric et al., 2010); crN-CC (residues 1-503) and the crN-CC[F145E] were cloned into pGEX6p-1 vector encoding for N-terminal GST-tags.

For protein preparation of the crN and crN-6HR[F145E] fragments, *Escherichia coli* BL21(DE3) (Stratagene) was used for protein production in LB media containing 40 μg/ml kanamycin. After growth to an OD_600_ of 0.6 at 37°C the cells were cooled to 20°C and expression was induced with 0.4 mM isopropyl 1-thio-β-galactopyranoside (IPTG). Protein expression was performed at 20°C for 16 hr.

Proteins were purified by immobilized metal-affinity chromatography (IMAC) on HisTrap HP Ni^2+^-Sepharose columns (GE Healthcare) at 4°C according to manufacturer's information. The hexahistidine tag was cleaved during dialysis against thrombin cleavage buffer (20 mM Tris-HCl, pH 7.4 supplemented with 150 mM NaCl and 2.5 mM CaCl_2_) for 16h at 4°C using 2 units of human thrombin (Sigma) per milligram of recombinant protein. Cleaved samples were reapplied to IMAC to separate the cleaved products from the hexahistidine tag and tagged proteins, concentrated and gel filtrated on a SEC HiLoad Superdex 200 16/60 column (GE Healthcare) equilibrated in 20 mM Tris-HCl, pH 7.5 supplemented with 150 mM NaCl and 2 mM DTT.

The recombinant protein expression of the crN-CC fragment was performed in *Escherichia coli* strain BL21 gold (DE3) in LB medium. Protein expression was induced at 18°C by addition of 0.4 mM IPTG and allowed to proceed for 18h. Cell pellets were lysed by lysozyme treatment and sonication, resuspended in lysis buffer containing 50 mM Tris-HCl (pH 7.5), 500 mM NaCl, 5 mM EDTA, 1 mM DTT, 1:1000 protease inhibitor cocktail (Sigma) and 0.5% Triton X-100. The lysates were incubated with Glutathion sepharose beads (GE healthcare). The beads were then washed ten times with lysis buffer supplemented with additional 500 mM NaCl. Proteins were eluted from the beads by removal of the GST-tags by prescission protease (GE healthcare) in a buffer containing 20 mM Tris-HCl (pH 7.5), 150 mM NaCl, 0.5 mM EDTA, 1 mM DTT, followed by size exclusion chromatography in 20 mM Tris-HCl (pH 7.5) supplemented with 150 mM NaCl and 2 mM DTT. Proteins were concentrated by centrifugal ultrafiltration.

The homogeneity of the recombinant proteins was assessed by SDS-PAGE and their identity confirmed by mass spectral analysis. Concentration of protein samples was determined by absorption at 280 nm.

**Structure Determination**

**C. elegans *SAS-6 ceN-Dimer***

Screening of crystallization conditions was performed using a Phoenix robot (Art Robin Instruments). Crystals of the ceN[S123E] variant were obtained at 4°C using the sitting drop vapor diffusion method. Drops of 1:1 mixture of ceN[S123E] at 10-15 mg/ml concentration and mother liquor (0.1 M MES, pH 6.0, 30% v/v PEG 200, 5% w/v PEG 3000) yielded crystals after 2-3 days. Initial crystal morphology was of irregularly stacked tetragonal plates; however, these crystals produced only weak powder-like diffraction patterns. We performed seeding experiments under the same conditions by crushing these crystals in mother liquor, and mixing the resulting seeds in 1:20-1:50 ratios with fresh protein just prior to crystallization drop setups under the same conditions. We obtained single, trigonal plate crystals that grew to a maximum size of ∼200 μm and maximum thickness of ∼40 μm. The mother liquor served as cryoprotectant. Heavy atom derivatives were prepared by over-night soaking of crystals in mother liquor supplemented with 10 mM NdCl_3_.

Diffraction data were collected under cryogenic conditions at the X06DA macromolecular crystallography beamline of the Swiss Light Source (SLS), Villigen PSI. Reflection data were indexed by LABELIT (Sauter et al., 2004), refined and integrated in XDS (Kabsch, 1993), and merged by SCALA (Evans, 2006). The Laue group and space group were suggested by POINTLESS (Evans, 2006) from the unmerged data, and data quality was assessed by PHENIX.xtriage (Adams et al., 2002). Native crystals diffracted to 2.1 Å resolution at a wavelength of 1 Å and belonged to the P2_1_2_1_2_1_ space group with a = 70.27 Å, b = 73.15 Å and c = 79.60 Å. The Matthews coefficient strongly suggested two protein molecules per asymmetric unit.

Phase information was obtained from a highly redundant dataset with maximum resolution of 3.1 Å collected on NdCl_3_ derivatized crystals at a wavelength of 1.6 Å. Phasing by SAD was performed using PHENIX.autosol (Adams et al., 2002) which located and refined 5 Nd sites to produce a density map with initial figure of merit of 0.44. Initial model building was done with PHENIX.autosol (209 residues built, 56 residues identified).

Manual building with COOT (Emsley and Cowtan, 2004) and refinement against the native data using PHENIX.refine (Adams et al., 2002) resulted in a final model with satisfactory R-work/R-free and MolProbity (Davis et al., 2007) statistics (Table S1). The ceN-dimer interface was analyzed using PDBePISA (Krissinel and Henrick, 2007).

**C. reinhardtii *Bld12p crN-Dimer and crCC-Dimer***

Crystallization was performed using the sitting-drop vapor-diffusion method. Pipetting was carried out on a Phoenix liquid handling robot (Art Robbins Instruments). The crN fragment was concentrated to 10 mg/ml and crN-6HR[F145E] to 15 mg/ml. Initial crystal hits were screened by in situ X-ray diffraction at beamline X06DA of the Swiss Light Source (SLS), Villigen PSI (Bingel-Erlenmeyer et al., 2011). crN crystals grew in 100 mM HEPES, pH 7.0, 20% PEG4000 at 20°C; crN-6HR[F145E] crystals grew as needles 400 μm long for 10-15 μm width in 100 mM TrisHCl, pH 8.5, 200 mM MgCl2, 20% PEG8000, 2% benzamidine at 20°C.

Data were collected at both X06SA (microdiffractometer) and X06DA beamlines of the Swiss Light Source (SLS), Villigen PSI, and processed with XDS (Kabsch, 1993). The N structure was solved by molecular replacement with Phaser (McCoy et al., 2007) using a truncated search model of the ceN[S123E] structure (see above) of *C. elegans* SAS-6. The refined structure of crN was used subsequently as a model for solving the structure of crN-6HR[F145E]. Refinement was done with either Phenix (Adams et al., 2002) or BUSTER (Blanc et al., 2004) and iterative model building with Coot (Emsley and Cowtan, 2004). Model statistics were obtained with MolProbity (Chen et al., 2010). Molecular visualizations and structure illustrations were carried out with Pymol (DeLano, 2002). Data processing and refinement statistics are summarized in Table S1.

**Plasmids for Human Cell Experiments**

The pEBTet-GFP plasmids (Bach et al., 2007) were obtained from Dirk Gründemann. The following oligos were annealed GW-F (CGCGGGTACCGCCGGCAGCTAGCGGCGCGCCCGGCCGATAT), GW-R (ATATCGGCCGGGCGCGCCGCTAGCTGCCGGCGGTACCCGCG), digested with KpnI and EagI and ligated into KpnI, NotI cut pEBTet-GFP producing the pEBTet-MCS vector. This plasmid was then used to insert fluorescence proteins and Gateway cassette (Invitrogen), generating the destination vector pEBTet-GW-EGFP. The multiple cloning site of pENTR 1A (Invitrogen) was modified by introducing single restriction sites between the attR1 and attR2 sites (3′-AgeI and XbaI-5′), generating the entry vector pENTR-SD-Age-AGT. Full length HsSAS-6 was amplified using the primers Age-Ko-HsSAS6-F (CGCGACCGGTACCATGAGCCAAGTGCTGTTCCAC) and Xba-noST-S6-R (CGCGTCTAG ATAACTGTTTGGTAACTGCCCA), and cloned into pENTR-SD-Age vector by restriction digest with AgeI and XbaI.

Mutations of the F131 residue in HsSAS-6 were performed by site-directed mutagenesis on pENTR-SD-Age-HsSAS-6 using the following primers: S6-F131E-fwd, *GAG*AAGCATCTTACACACCTCTCAC and S6-F131R-fwd, *CGA*AAGCATCTTACACACCTCTCAC (mutated codon is italicized).

Gateway reaction was then performed according to the manufacturer's protocol to generate the expression plasmid pEBTet-HsSAS-6-GFP and pEBTet-HsSAS-6-F131E/R-GFP, which were sequence verified.

## Accession Numbers

Coordinates have been deposited in the Protein Data Bank with accession codes 3PYI (ceN), 3Q0Y (crN), and 3Q0X (crCC).

## Supplemental Information

Table S1. X-Ray Data Collection and Refinement Statistics

Movie S1. Time-Lapse DIC Microscopy of *sas-6(RNAi)* Embryos, Related to Figure 3 Monitored from the one-cell stage until the end of the second cell cycle. Images were captured every 5 s, and the movie is played at 10 frames per second. Embryos are ∼50 mm long and oriented with anterior to the left; time is indicated in minutes:seconds.

Movie S2. Time-Lapse DIC Microscopy of *sas-6(RNAi)* Embryos Expressing GFP-SAS-6RR, Related to Figure 3 Monitored from the one-cell stage until the end of the second cell cycle. Images were captured every 5 s, and the movie is played at 10 frames per second. Embryos are ∼50 μm long and oriented with anterior to the left; time is indicated in minutes:seconds.

Movie S3. Time-Lapse DIC Microscopy of *sas-6(RNAi)* Embryos Expressing GFP-SAS-6RR[I154E], Related to Figure 3 Monitored from the one-cell stage until the end of the second cell cycle. Images were captured every 5 s, and the movie is played at 10 frames per second. Embryos are ∼50 μm long and oriented with anterior to the left; time is indicated in minutes:seconds.

Document S1. Article Plus Supplemental Information
