# Supplementary figures and images for: Structural Basis of the 9-Fold Symmetry of Centrioles
Source: Cell. 2011 Feb 4;144(3):364–75. doi: 10.1016/j.cell.2011.01.008 (PMC3089914; doi:10.1016/j.cell.2011.01.008)

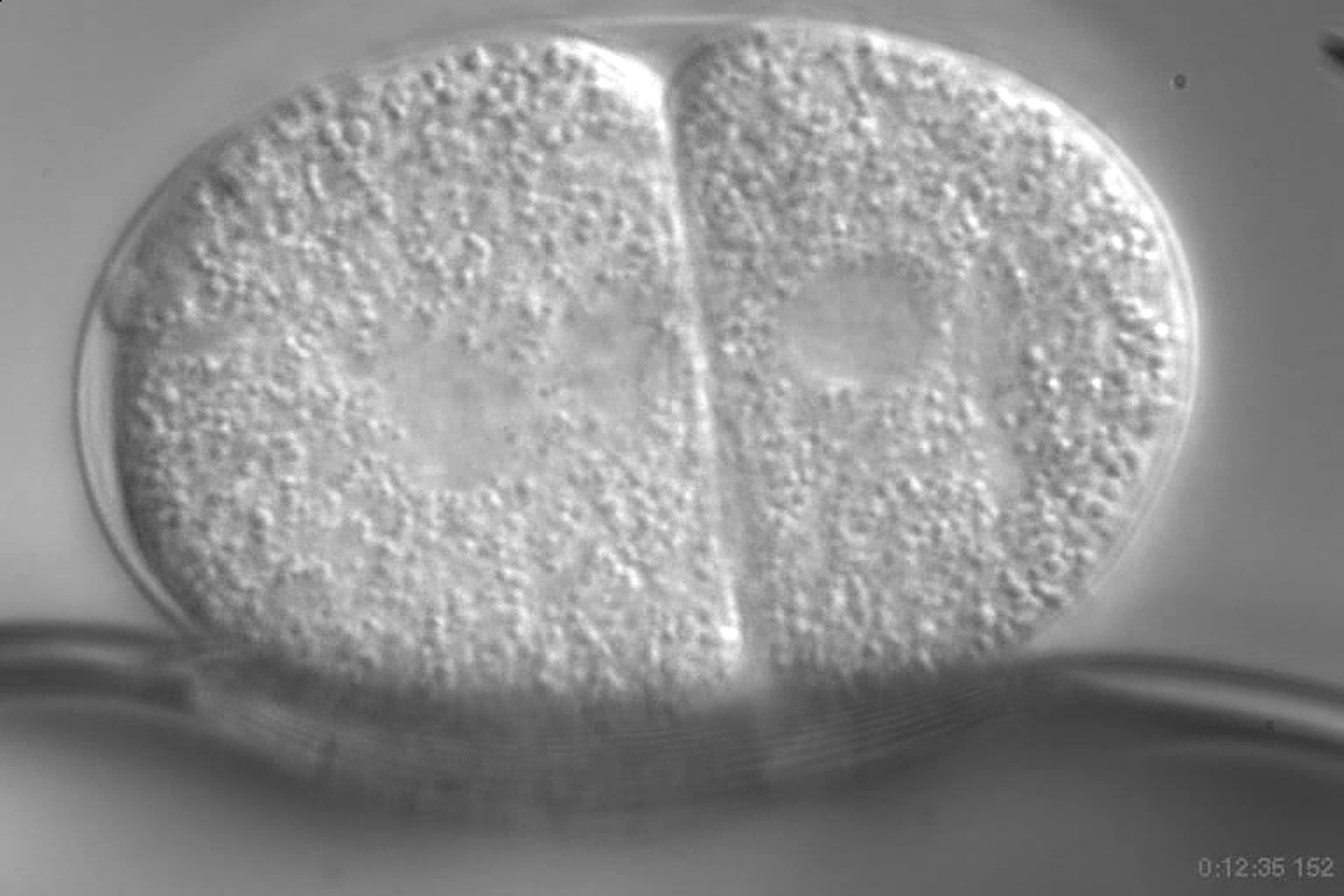

Supplement: Movie S1. Time-Lapse DIC Microscopy of sas-6(RNAi) Embryos, Related to Figure 3 — Monitored from the one-cell stage until the end of the second cell cycle. Images were captured every 5 s, and the movie is played at 10 frames per second. Embryos are ∼50 mm long and oriented with anterior to the left; time is indicated in minutes:seconds. [file mmc2.jpg]

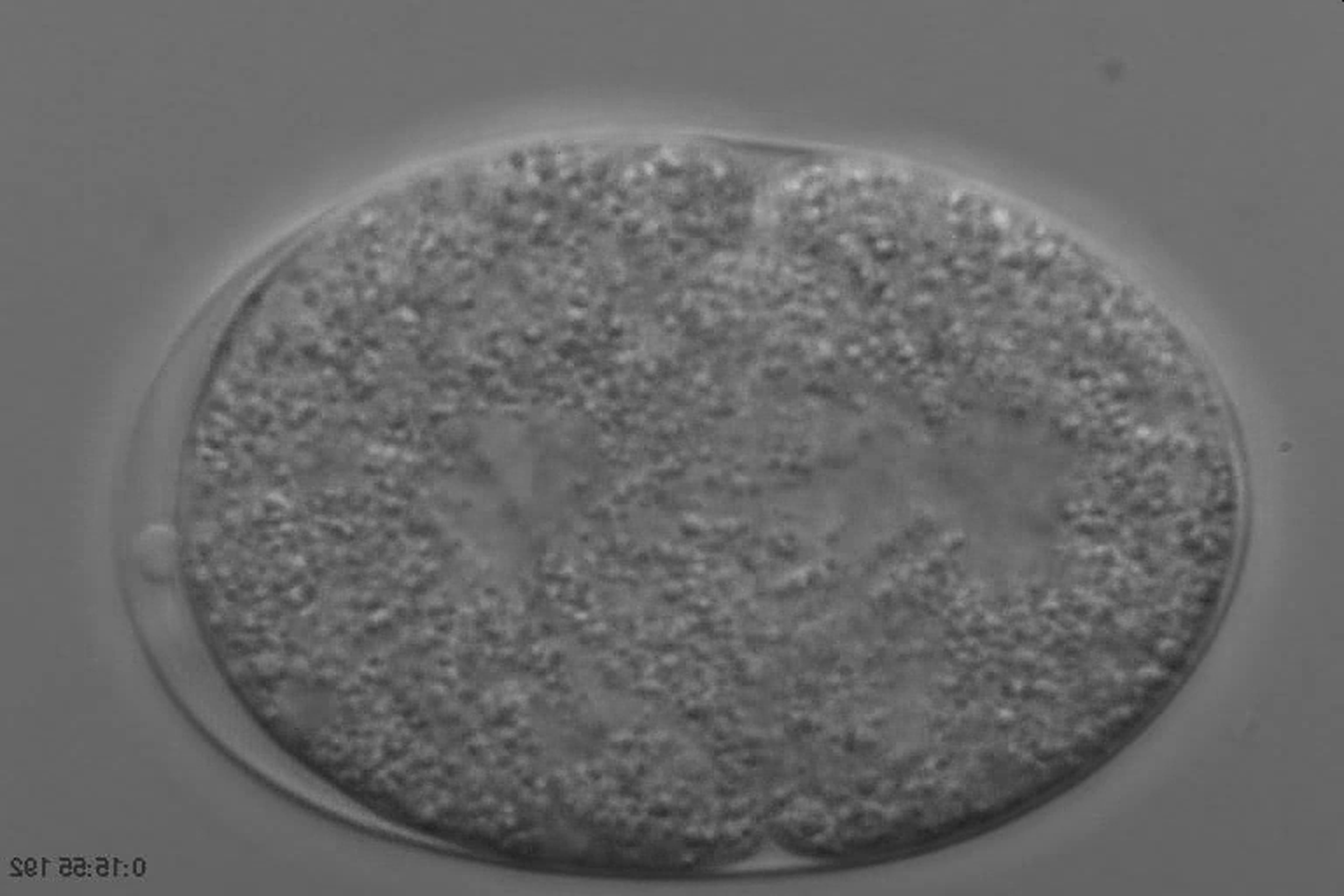

Supplement: Movie S2. Time-Lapse DIC Microscopy of sas-6(RNAi) Embryos Expressing GFP-SAS-6RR, Related to Figure 3 — Monitored from the one-cell stage until the end of the second cell cycle. Images were captured every 5 s, and the movie is played at 10 frames per second. Embryos are ∼50 μm long and oriented with anterior to the left; time is indicated in minutes:seconds. [file mmc3.jpg]

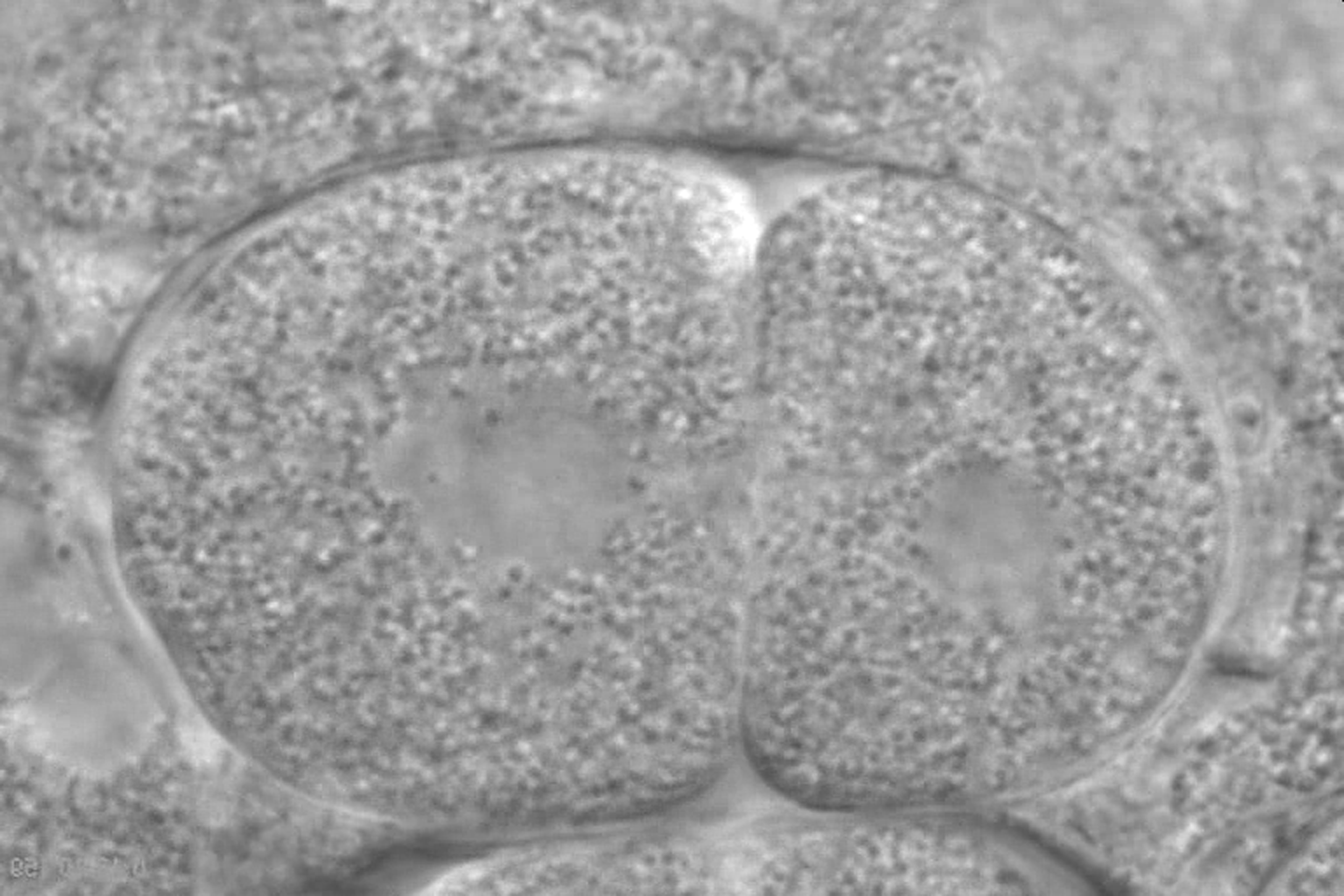

Supplement: Movie S3. Time-Lapse DIC Microscopy of sas-6(RNAi) Embryos Expressing GFP-SAS-6RR[I154E], Related to Figure 3 — Monitored from the one-cell stage until the end of the second cell cycle. Images were captured every 5 s, and the movie is played at 10 frames per second. Embryos are ∼50 μm long and oriented with anterior to the left; time is indicated in minutes:seconds. [file mmc4.jpg]
